# Tumor suppressor p53 protects mice against *Listeria monocytogenes* infection

**DOI:** 10.1038/srep33815

**Published:** 2016-09-20

**Authors:** Shaohui Wang, Pingping Liu, Jianchao Wei, Zixiang Zhu, Zixue Shi, Donghua Shao, Zhiyong Ma

**Affiliations:** 1 Shanghai Veterinary Research Institute, Chinese Academy of Agricultural Sciences, No. 518, Ziyue Road, Shanghai 200241, China; 2 State Key Laboratory of Veterinary Etiological Biology, Lanzhou Veterinary Research Institute, Chinese Academy of Agricultural Sciences, Lanzhou 730046, China

## Abstract

Tumor suppressor p53 is involved in regulating immune responses, which contribute to antitumor and antiviral activity. However, whether p53 has anti-bacterial functions remains unclear. *Listeria monocytogenes* (LM) causes listeriosis in humans and animals, and it is a powerful model for studying innate and adaptive immunity. In the present study, we illustrate an important regulatory role of p53 during LM infection. p53 knockout (p53KO) mice were more susceptible to LM infection, which was manifested by a shorter survival time and lower survival rate. p53KO mice showed significant impairments in LM eradication. Knockdown of p53 in RAW264.7 and HeLa cells resulted in increased invasion and intracellular survival of LM. Furthermore, the invasion and intracellular survival of LM was inhibited in p53-overexpressing RAW264.7 and HeLa cells. LM-infected p53KO mice exhibited severe clinical symptoms and organ injury, presumably because of the abnormal production of the pro-inflammatory cytokines TNF-α, IL-6, IL-12, and IL-18. Decreased IFN-γ and GBP1 productions were observed in LM-infected p53-deficient mice or cells. The combination of these defects likely resulted in the overwhelming LM infection in the p53KO mice. These observations indicate that p53 serves as an important regulator of the host innate immune that protects against LM infection.

*Listeria monocytogenes* (LM) is a Gram-positive bacterium that often causes invasive diseases in humans and animals, especially central nervous system infections. Immunocompromised individuals, including newborns and elderly people, are particularly vulnerable to LM infection, which can lead to septicemia and meningitis, whereas LM infection of pregnant woman can occasionally lead to septic abortion. LM is a food-borne pathogen that can survive and grow in extreme conditions. Humans are exposed to LM by ingesting contaminated foods, such as unpasteurized dairy products and incompletely cooked meats^1^.

LM is often used to study the mammalian immune response to infection^2^,^3^,^4^,^5^,^6^. Following infection with LM, innate immune responses are rapidly triggered, and they are essential for early control of LM infection. Macrophages are the primary host cells for LM *in vivo*, since LM replication occurs within them. Macrophages have been the focus of innate immunity during LM infection, because they were thought to be the principal mediators of the killing of LM. Mice whose granulocytes were depleted using antibodies showed significantly enhanced susceptibility to LM infection^7^,^8^,^9^,^10^. In response to LM infection, macrophages secrete tumor-necrosis factor (TNF)-α and interleukin (IL)-12. These cytokines drive natural killer cells to produce interferon-γ (IFN-γ), which in turn activates macrophages and increases their bactericidal activity. IFN-γ, TNF-α, and other pro-inflammatory cytokines may be the most important cytokines for controlling LM infection, and mice lacking these cytokines or their cognate receptors are highly susceptible to LM infection^11^,^12^,^13^,^14^,^15^,^16^,^17^,^18^.

Tumor suppressor p53 plays an important role in the regulation of various biological processes, such as cell cycle arrest, DNA repair, and apoptosis. p53 primarily functions as a transcription factor that regulates the expression of target genes, which play important roles in biological processes^19^,^20^. For instance, p53 transactivates the expression of its target gene p21 to arrest the cell cycle, as well as its target gene retinoic acid-inducible gene I to regulate cell survival and growth^21^,^22^. p53, which acts as a suppressor of the inflammatory response, plays a role in the innate immune response *in vivo*, which is consistent with its tumor suppressor function^23^. Recently, p53 has been shown to contribute to the host immune response against viral infections, such as vesicular stomatitis virus, Newcastle disease virus, and hepatitis C virus^24^,^25^,^26^,^27^,^28^. In addition, p53 could integrate host defense and cell fate to against the infection of Gram-negative extracellular bacteria *Klebsiella pneumonia*^29^. However, the role of p53 in protection against intracellular bacteria LM infection is less clear.

In this study, we addressed the roles of p53 in resisting LM infection. Interestingly, mice lacking p53 showed marked susceptibility to LM as evidenced by delayed bacterial clearance, elevated numbers of circulating neutrophils, and differential expression patterns of pro-inflammation cytokines. These observations indicate that p53 serves as an important regulator of the host innate immune that protects against LM infection.

## Results

### p53 is required for resistance to LM infection

**Figure 1:**
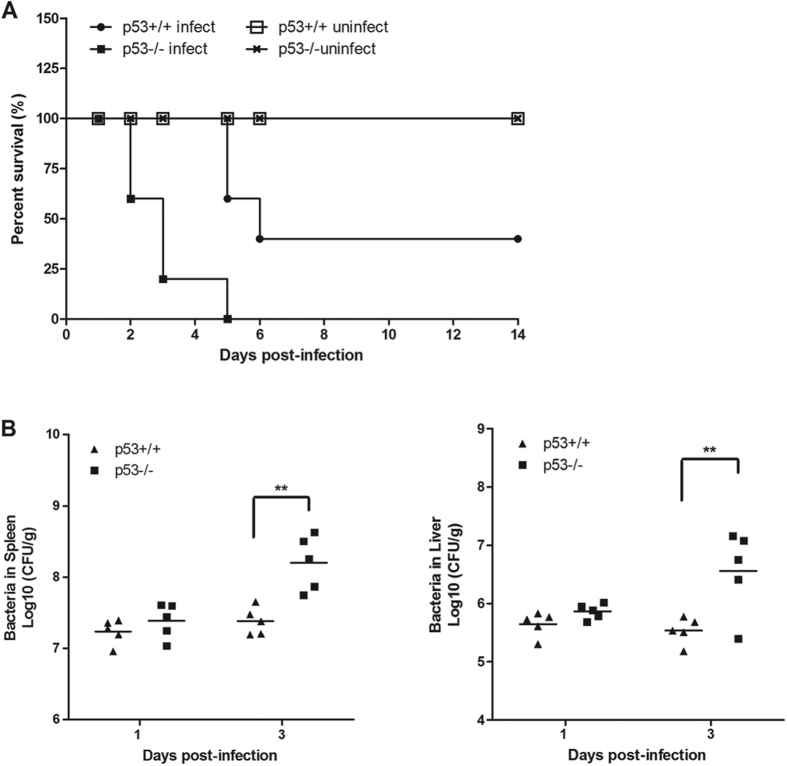
p53KO mice are more susceptible to LM infection than p53WT mice. (**A**) p53KO mice die more rapidly than p53WT mice. Mice were infected intra-peritoneally with 1 × 10^6^ colony-forming units (CFUs) of LM strain 10403 s. Ten mice per experimental group were monitored for survival for 14 d. (**B**) Clearance of LM infection is impaired in p53KO mice. p53WT and p53KO mice were infected intra-peritoneally with 1 × 10^5^ CFUs of LM strain 10403 s. Mice were sacrificed on day 1 and 3 post-infection, and the bacterial loads in the livers and spleens were determined. Horizontal bars indicate the means for each sample group. Statistical analyses were performed using a Mann–Whitney test. Statistically significant differences compared with the control group are indicated by asterisks (**P < 0.01).

To assess the role of p53 during LM infection, we compared the lethality of LM to p53 wild-type (p53WT) mice and p53 knockout (p53KO) mice. Ten 8-week-old p53WT and p53KO mice were infected intra-peritoneally with LM strain 10403s. Mock-infected mice that were treated with phosphate-buffered saline (PBS) were used as a negative control. The results showed that p53KO mice died at 2 d post-infection, whereas p53WT mice died at 5 d post-infection. All of the p53KO mice died at 5 d post-infection, and only 60% the p53WT mice died at 6 d post-infection. An accelerated and increased lethality was revealed in the p53KO mice (Fig. 1A). This result indicates that p53 is essential for the host to protect against LM infection.

### Critical role of p53 in eradication of LM in the spleen and liver

Next, we examined the role of p53 in the clearance of LM during infection. The bacterial loads in the livers and spleens of p53WT and p53KO mice inoculated with a sub-lethal dose of LM were determined. As expected, the p53KO mice showed an impairment in LM eradication, compared with the p53WT mice. At 1 d post-infection, the bacterial numbers in the liver and spleen of the p53KO mice were slightly greater than those of the p53WT mice (*P* > 0.05). Moreover, the p53KO mice exhibited significantly increased bacterial loads in both the liver and spleen after 3 d post-infection, compared with the p53WT mice (*P* < 0.01) (Fig. 1B). These results indicate that p53 plays an essential role in eradicating LM from the spleen and liver.

### Loss of p53 leads to severe pathological damage in LM-infected mice

**Figure 2:**
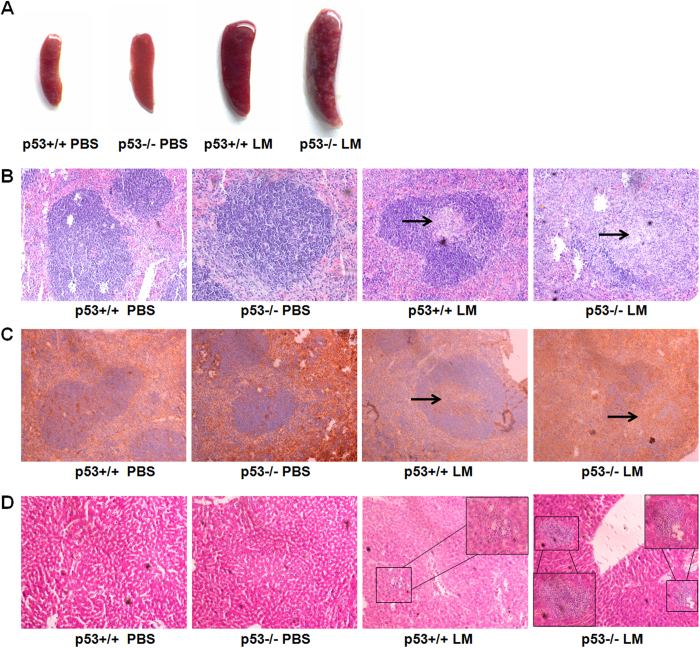
Increased pathological damage in p53KO mice, compared with p53WT mice. At 3 d post-infection, mice were sacrificed, and pathological changes were observed. (**A**) Clinical examination of mice infected with LM. p53KO mice displayed more severe pathological damage in the spleen than p53WT mice. (**B**) Spleen sections of LM-infected mice were stained with hematoxylin-eosin (HE). The p53KO mice showed more severe lymphocyte depletion within white pulp follicles of the spleen than the p53WT mice. Arrows point to lymphocyte depletion. (**C**) Spleen sections of LM-infected mice were stained with an anti-CD3 monoclonal antibody. T lymphocyte depletion was severe in the LM-infected p53KO mice, while it was weak in the p53WT mice. Arrows point to T lymphocyte depletion. (**D**) Liver sections of LM-infected mice were stained with hematoxylin-eosin (HE). The number and area of inflammatory infiltrate foci of LM-infected p53KO were greater than those of the p53WT mice. There were no pathological changes in the spleen and liver tissues of the control group.

We further investigated the pathological changes in the livers and spleens of LM-infected mice. p53KO and p53WT mice were infected with a sub-lethal dose of LM. At 1 d post-infection, there were no noticeable tissue lesions in the p53KO and p53WT mice (data not shown). On day 3 post-infection, listeriosis had progressed in both groups of mice. Consistent with the bacterial loads, mice lacking p53 showed more severe pathological damage than the p53WT control mice. The results showed that the spleens of the p53KO mice displayed severe tumefaction, compared with those of p53WT mice (Fig. 2A). However, there were no remarkable, visible differences in the pathological changes of the liver between the p53WT and p53KO mice. Moreover, the spleens from the p53KO mice displayed increased lymphocyte depletion, compared with those of the p53WT mice (Fig. 2B). In addition, staining with a rat anti-mouse CD3 monoclonal antibody further validated the T lymphocyte depletion (Fig. 2C). Inflammatory infiltrate foci, consisting of numerous neutrophils, in the liver of LM-infected mice correlated with the bacteria burden. Similarly, the number and area of inflammatory infiltrates in the livers of the p53KO were greater than those of the p53WT mice (Fig. 2D)^30^,^31^. These results may reflect the impaired resistance to LM infection in the p53KO mice.

### p53 inhibits LM invasion of RAW264.7 and HeLa cells

**Figure 3:**
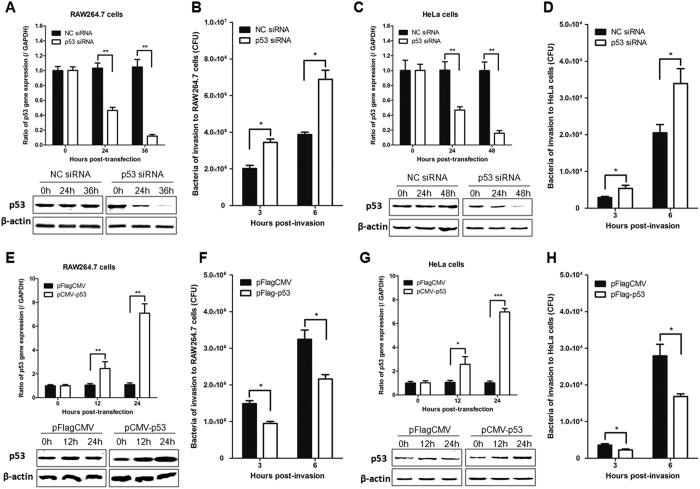
p53 plays a protective role against LM invasion *in vitro*. (**A–D**) Knockdown of p53 facilitates the invasion of LM into RAW264.7 and HeLa cells. RAW264.7 and HeLa cells were transfected with a p53 siRNA or a negative control siRNA, and the p53 expression levels were determined by qRT-PCR and western blotting (**A,C** respectively). Knockdown of p53 led to significantly increased invasion and intracellular survival capacities in RAW264.7 cells (**B**) and HeLa cells (**D**). (**E–H**) Overexpression of p53 inhibits LM invasion of RAW264.7 and HeLa cells. RAW264.7 and HeLa cells were transfected with pFLAG-p53, which expresses FLAG-p53, or a control vector (pFLAG-CMV). The p53 expression levels were determined by qRT-PCR and western blotting (**E,G** respectively). Overexpression of p53 resulted in significantly reduced bacterial invasion capacities, compared with that of control cells (**F,H**). Statistically significant differences compared with the control group are indicated by asterisks (*P < 0.05; **P < 0.01; ***P < 0.001).

Next, we assessed whether p53 protects against infection *in vitro*. We used different species of small interfering RNAs (siRNAs) to knockdown p53 expression in mouse macrophage-like RAW264.7 cells and HeLa cells. As a control, cells were transfected with a negative control siRNA, which is a non-targeting siRNA. The results showed that silencing of p53 expression levels was robust and gene-specific, as confirmed by quantitative real-time reverse transcription-polymerase chain reaction (qRT-PCR) and western blotting (Fig. 3A,C). After transfection, cells were infected with LM, and the invasion and intracellular survival of LM were compared. For the same multiplicity of infection (MOI), knockdown of p53 expression resulted in significantly increased invasion efficiencies and intracellular survival of LM, compared with control cells (*P* < 0.05) (Fig. 3B,D).

In addition, we transiently overexpressed p53 to determine whether it had an inhibitory effect on LM invasion and intracellular survival. RAW264.7 and HeLa cells were transfected with a plasmid expressing FLAG-tagged p53 (FLAG-p53) and subsequently infected with LM. The expression of p53 was confirmed by qRT-PCR and western blotting (Fig. 3E,G). During infection with LM, the stable overexpression of p53 resulted in significantly reduced bacteria invasion, compared with that of control cells (*P* < 0.05) (Fig. 3F,H). These observations further confirm that p53 plays a role in protection against LM infection.

### Impaired IFN-γ and guanylate-binding protein 1 (GBP1) production in LM-infected p53-deficient mice/cells

**Figure 4:**
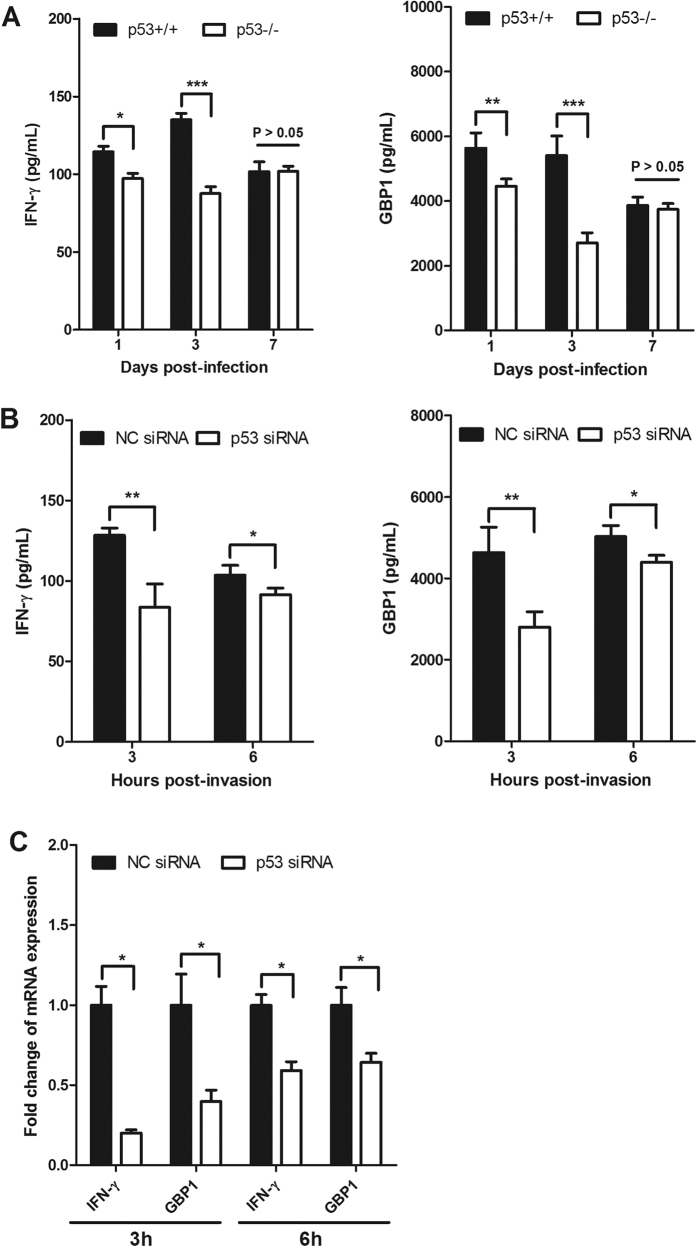
Analysis of IFN-γ and GBP1 expression in p53-deficient mice/cells that were infected with LM. (**A**) Mice were infected with LM for the times indicated, and the production of IFN-γ and GBP1 in serum was determined by ELISA. p53KO mice exhibit reductions of IFN-γ and GBP1 in serum in response to LM infection. Data represent the average of five mice per group per time point. (**B**) RAW264.7 cells were transfected with a p53 siRNA or a non-coding siRNA, and then they were infected with LM at MOI of 10. The supernatant was collected and the concentrations of IFN-γ and GBP1 were determined by ELISA. Knockdown of p53 expression led to decreased IFN-γ and GBP1 expression levels in LM-infected RAW264.7 cells. (**C**) A p53 siRNA or a non-coding siRNA were transfected into RAW264.7 cells, which were subsequently infected with LM. At the indicated times, the cells were collected and the IFN-γ and GBP1 transcription levels were measured by qRT-PCR. Knockdown of p53 expression significantly decreased the IFN-γ and GBP1 transcription levels. Statistically significant differences compared with the control group are indicated by asterisks (*P < 0.05; **P < 0.01; ***P < 0.001).

Considerable work has demonstrated that IFN-γ protects against LM infection^10^,^17^,^18^. GBP1 is an IFN-γ-inducible protein that is involved in the host immune response against LM infection^32^. Thus, we monitored the serum concentrations of IFN-γ and GBP1 in p53WT and p53KO mice that were infected with LM. The results showed that the proportions of IFN-γ and GBP1 significantly decreased in the absence of p53 as early as 1 d post-infection (*P* < 0.05). Furthermore, mice lacking p53 showed noticeably diminished levels of IFN-γ and GBP1 through the 3 d course of infection (*P* < 0.001). However, the levels of IFN-γ and GBP1 in both mice were similar on 7 d post-infection (*P* > 0.05). In addition, the expression levels of IFN-γ and GBP1 in LM-infected RAW264.7 cells were determined by enzyme-linked immunosorbent assays (ELISA) and RT-PCR. Consistent with the reduced IFN-γ and GBP1 concentrations in p53KO mice, knockdown of p53 expression significantly decreased the expression levels of IFN-γ and GBP1 during infection for the indicated times (*P* < 0.05 and *P* < 0.01, respectively) (Fig. 4). The transcription levels correlated with the expression levels, as p53 knockdown cells showed reduced transcription levels of IFN-γ and GBP1, compared with those of the control group. These results suggest that p53 plays an essential role in the clearance of LM via activation of IFN-γ and GBP1.

### Pro-inflammatory cytokine responses are misregulated during LM infection in p53-deficient mice

**Figure 5:**
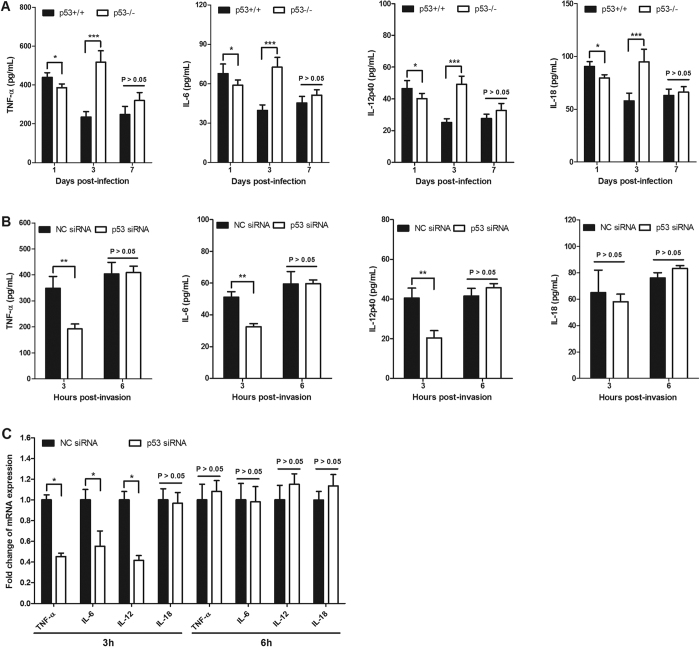
Analysis of the pro-inflammatory cytokines TNF-α, IL-6, IL-12 and IL-18 in p53-deficient mice/cells that were infected with LM. (**A**) Mice were infected with LM for the times indicated, and the production of TNF-α, IL-6, IL-12, and IL-18 in serum was quantified by ELISA. Data represent the average from five mice per group per time point. (**B**) RAW264.7 cells were transfected with a p53 siRNA or a non-coding siRNA, followed by infection with LM at MOI of 10. The supernatant was collected and the TNF-α, IL-6, IL-12, and IL-18 concentrations were determined by ELISA. (**C**) RAW264.7 cells were transfected with a p53 siRNA or a non-coding siRNA, and then they were subsequently infected with LM. At the indicated times, the cells were collected and the TNF-α, IL-6, IL-12, and IL-18 transcription levels were measured by qRT-PCR. Statistically significant differences compared with the control group are indicated by asterisks (*P < 0.05; **P < 0.01; ***P < 0.001).

A previous study indicated that p53 acts as a suppressor of the inflammatory response, and an abnormal inflammatory response, which was followed by severe organ damage, was found in p53KO mice^23^. To determine whether p53 affects pro-inflammatory cytokine production, we quantified the levels of several cytokines in the serum of p53KO mice that were infected with LM. The major pro-inflammatory cytokines TNF-α, IL-6, IL-12, and IL-18 play key roles in determining the inflammatory response^15^,^16^,^23^. Moreover, these pro-inflammatory cytokines are essential for early clearance of LM during infection. Unexpectedly, the serum concentrations of TNF-α, IL-6, IL-12, and IL-18 were significantly lower in the p53KO mice than in the p53WT mice at 1 d post-infection (*P* < 0.05). However, the lack of p53 led to significantly elevated concentrations of these pro-inflammatory cytokines at 3 d post-infection (*P* < 0.001), which likely reflects the increased bacterial burden and inflammatory response. At the later stage of infection, the production of pro-inflammatory cytokines by the p53KO mice was similar to that of the p53WT mice. Macrophages are the primary produces of pro-inflammatory cytokines. Thus, we examined the contribution of p53 to the functions of RAW264.7 cells. As shown in Fig. 5B, knockdown of p53 expression in RAW264.7 cells resulted in significantly lower TNF-α, IL-6, and IL-12 levels, compared with those of control cells, after 3 h of LM invasion. By contrast, the production of IL-18 was not significantly affected by the p53 deficiency. In line with the expression levels, the TNF-α, IL-6, and IL-12 transcription levels were significantly decreased by the knockdown of p53 expression. However, after 6 h of invasion, the transcription and expression levels of TNF-α, IL-6, and IL-12 were similar in both cells.

## Discussion

This study illustrates an important regulatory role of p53 during LM infection. We found that p53KO mice were highly susceptible to LM infection. This was manifested by a shorter survival time and lower survival rate. After the LM infection, p53KO mice were less efficient in controlling bacterial replication, and they exhibited more severe pathological damage. Moreover, knockdown of p53 expression in RAW264.7 and HeLa cells led to increased susceptible to LM infection. These phenomena might be due to the impaired production of IFN-γ and GBP1, as well as an abnormal inflammatory response, in p53KO mice.

It has been shown that LM infection can trigger an extensive host innate immune response. In fact, LM replicates intracellularly, and the number of bacteria is limited by activated macrophages, which prevent the dissemination of LM into the bloodstream^7^,^8^,^9^,^10^. LM-infected macrophages produce the pro-inflammatory cytokines TNF-α, IL-6, IL-12, and IFN-γ, which subsequently activate macrophages and neutrophils to kill LM via the production of listericidal molecules, such as nitric oxide^11^,^12^,^13^,^14^,^15^,^16^,^17^,^18^. Recruitment of microbicidal neutrophils are required for the host to successfully fight a bacterial infection. However, over-active inflammation is harmful to the host because it can lead to organ damage^33^. In the present study, we observed severe lymphocyte depletion and complete destruction of all white pulp follicles in the spleen of p53KO mice, whereas these symptoms were not observed in the p53WT group. In addition, p53KO mice showed an accumulation of neutrophils in filtrates in the liver. Indeed, p53KO exhibited increased macrophage recruitment and delayed neutrophil clearance in response to inflammation-inducing agents, compared with p53WT mice. Interestingly, delayed neutrophil clearance was accompanied by accumulation of inflammatory cells in the liver of p53KO mice, presumably due to higher levels of pro-inflammatory cytokines^23^,^34^. Moreover, the survival of mice with different p53 genotypes was consistent with the observed organ damage, which might have caused the deaths of the p53KO mice. Although lung injury was not associated with impaired bacterial clearance, p53KO mice showed lower survival rates when infected with *Klebsiella pneumoniae*^29^.

The pro-inflammatory cytokines TNF-α, IL-6, and IL-12 act as early signals during innate immunity, and they are critical for LM clearance^15^,^16^. Therefore, we investigated the production and expression patterns of these cytokines in response to LM infection. In the present study, significant upregulated expressions of TNF-α, IL-6, IL-12, and IL-18 were observed in p53WT mice at the initial stage of LM infection. Subsequently, at the later time points (3 d and 7 d), the production of TNF-α, IL-6, IL-12, and IL-18 returned to moderate levels in LM-infected p53WT mice. Unexpectedly, the expression of TNF-α, IL-6, IL-12, and IL-18 was significantly reduced in p53KO mice at 1 d post-infection (*P* < 0.05). However, a previous study indicated that p53 deletion led to the induction of pro-inflammatory genes^29^. TNF-α, IL-6, IL-12, and IL-18 are essential for recruitment of innate immune cells at early time points. During infection, a host utilizes different the innate immune response, including pro-inflammatory cytokines, to defend against LM infection. Although p53 can inhibit the inflammatory response in wild-type mice/cells, a host could effectively induce the expression of pro-inflammatory genes via other signaling pathways to ensure the early clearance of LM. In contrast, a previous study indicated that p53 may selectively affect the production of inflammatory cytokines^34^. This might be a reason for the inconsistent results regarding pro-inflammatory cytokine expression. However, p53KO mice exhibited significantly elevated levels of TNF-α, IL-6, IL-12, and IL-18 at a later stage of infection. It is known that TNF-α, IL-6, and IL-12 are important mediators of the inflammatory response. The release of these pro-inflammatory cytokines at the site of infection appears to play a key role in regulating inflammation, which contributes to tissue pathology and affects the disease outcome. Previous studies showed that p53KO mice exhibited higher mortality rates in response to LM infection because of abnormal inflammatory response, which was followed by severe organ damage and increased sensitivity to septic shock^23^,^34^. Similarly, heat shock factor 1 is needed to protect mice from rapid death during LM infection by preventing the overproduction of TNF-α^35^. Thus, the prolonged overproduction of pro-inflammatory cytokines may contribute to serious organ injury, which can rapidly lead to septic shock and the rapid death of p53KO mice. These results may explain why p53KO mice are susceptible to LM infection. This conclusion is consistent with a previous study that showed a significant number of early deaths of p53KO mice resulted from unresolved, spontaneous inflammation^36^.

It is well known that LM infection induces IFN-γ production and that IFN-γ is the most important cytokine for host defense against LM. In fact, IFN-γ constitutes a late pro-inflammatory cytokine, bridging the innate and adaptive immune response and facilitating the full clearance of LM from the host. Previous studies showed that IFN-γ-deficient mice exhibited increased susceptibility to LM infection due to the failure to generate an appropriate immune response^17^,^18^. IFN-γ induces resistance to bacterial infection through broad transcriptional programs involving a variety of genes, many of which remain uncharacterized. Guanylate-binding proteins (GBPs) are IFN-induced GTPase family proteins, which are involved in various biological processes, such as antitumor and antiviral immune responses^37^,^38^,^39^. A recent study indicated that IFN-γ-inducible GBP1 plays a critical role in resistance to intracellular pathogens (LM and *Mycobacterium bovis*). GBP1 coordinates a potent oxidative and vesicular trafficking program to protect the host from infection^32^. p53 acts as a transcription factor that regulates the expression of target genes, and our previous study indicated that GBP1 is a direct transcriptional target gene of p53. p53 positively regulates GBP1 expression by binding to the p53 response element in the GBP1 promoter region^40^. In the present study, we observed that the production of IFN-γ and GBP1 was significantly reduced in the absence of p53 after LM infection, suggesting an essential role of p53 in IFN-γ and GBP1 activation during cellular responses to LM infection.

In conclusion, our study demonstrates an important, previously undescribed role for p53 in defense against LM infection. Mice deficient in p53 phenotypically differed from p53WT mice in terms of disease progression, and they exhibited delayed bacterial clearance, an increase in the number of circulating neutrophils, and differential expression patterns of pro-inflammation cytokines. Each of these processes is required for LM resistance. Thus, the combination of these contributing factors ultimately resulted in an overwhelming infection and the rapid death of p53KO mice. These observations highlight the importance of p53 as an important regulator in the host innate immune that protects against LM, and they provide new insights into the function of p53 in LM infections.

## Methods

### Mice

Homozygous p53^+/+^ (p53WT) and p53^−/−^ (p53KO) mice were generated from breeding pairs of heterozygous p53^*+/*−^ C57BL/6 mice as reported previously^41^. Mice were cared for under specific-pathogen-free conditions in the Shanghai Veterinary Medicine Institute, Chinese Academy of Agricultural Sciences.

### Ethics statement

All animal experiments were conducted in strict accordance with the Guidelines on the Humane Treatment of Laboratory Animals (Ministry of Science and Technology of the People’s Republic of China, Policy No. 2006 398) and were approved by the Institutional Animal Care and Use Committee at the Shanghai Veterinary Research Institute (permit No: Shvri-Mo-0113).

### Bacteria and infection of mice

LM strain 10403s was grown to mid-log phase in brain-heart infusion (BHI) broth, and then washed and resuspended in sterile PBS. For bacterial infection, 8-week-old p53WT and p53KO mice (n = 10/group) were infected via intra-peritoneal injection with 1 × 10^6^ or 1 × 10^5^ CFUs of LM 10403s. Bacterial CFUs contained in the injected inoculum were confirmed by plating on BHI agar. Negative controls were injected with PBS. Mortality was monitored each day until 14 d post-infection.

### Enumeration of bacteria in the spleen and liver

Bacterial colonization of spleens and livers was determined as described previously, with some modification^42^. Briefly, mice inoculated with LM were euthanized and dissected at 1 and 3 d post-infection. Spleens and livers were harvested and homogenized in PBS containing 0.5% Triton X-100. Serial 10-fold dilutions were plated on BHI agar, and bacterial CFUs were assessed after overnight growth at 37 °C.

### Histological analysis

The histological changes of the spleen and liver were monitored at 1 and 3 d after challenge. Liver and spleen sections were fixed in buffered formaldehyde and embedded in paraffin. Paraffin sections were stained with hematoxylin-eosin (HE) and viewed by light microscopy.

For immunohistochemistry analysis, paraffin sections of spleen were stained by an indirect immunoperoxidase protocol using a rat anti-mouse CD3 monoclonal antibody (Abcam, Cambridge, MA, USA) as the primary antibody. Subsequently, sections were incubated with peroxidase-linked sheep anti-rat IgG (Amersham Biosciences, GE Healthcare, Little Chalfont, UK), followed by development with diaminobenzidine (Sigma-Aldrich, St. Louis, MO, USA).

### Cell transfection and bacterial infection

RAW264.7 and HeLa cells were cultured in Dulbecco’s modified Eagle’s medium (DMEM) (Gibco, Thermo Fisher Scientific, Waltham, MA, USA) without antibiotics at 37 °C under a 5% CO_2_ atmosphere. Wild-type p53 (mice and human) cDNAs were obtained and subcloned into the p3 × FLAG-CMV7.1 vector (Sigma-Aldrich) to generate a recombinant plasmid that overexpresses pFLAG-p53 as described previously^43^. For the knockdown of p53 expression, siRNAs specific for p53 (mice and human) were purchased from Shanghai GenePharma Co., Ltd. (Shanghai, China) (Table 1). Cells were transfected using Lipofectamine™ 2000 (Invitrogen, Thermo Fisher Scientific) according to the manufacturer’s protocol. The expression of p53 was determined by qRT-PCR and western blotting.

For bacterial infection, LM strain 10403s was grown to mid-log phase, and then washed and resuspended in DMEM without fetal bovine serum. After transfection, bacterial suspensions were added to monolayers at a MOI of 10 for RAW264.7 cells, or 100 for HeLa cells. At 1 h post-infection, the cells were washed and incubated with DMEM containing gentamicin (100 μg/mL) for 1 h to kill extracellular bacteria. Then, the cells were grown in DMEM containing 10 μg/mL gentamicin for an additional 3 or 6 h, followed washing and lysis in 0.5% Triton X-100 in PBS. Serial dilutions were plated on BHI agar overnight to determine the number of viable bacteria.

### Measurement of pro-inflammatory cytokine levels

Sera from LM-infected mice and the supernatant of LM-infected cells were collected at the indicated times. The concentrations of the pro-inflammatory cytokines TNF-α, IL-6, IL-12, IL-18, IFN-γ, and GBP1 were assayed simultaneously using an ELISA kit (BIOSAMITE, Shanghai, China) according to the manufacturer’s instructions.

### Quantitative real-time reverse transcription PCR (qRT-PCR)

Total RNA was extracted from cells using the TRIzol^®^ Reagent (Thermo Fisher Scientific) according to the manufacturer’s protocol. The quantity of total RNA was measured by a spectrophotometer (GE Healthcare). cDNA was synthesized using Moloney murine leukemia virus reverse transcriptase (Thermo Fisher Scientific). qRT-PCR was performed using SYBR Premix Ex Taq™ (Takara, Dalian, China) and gene-specific primers (Table 1) according to the manufacturer’s protocol. The relative gene expression was normalized to the expression of the housekeeping gene glyceraldehyde-3-phosphate dehydrogenase (GAPDH). PCR efficiency (>90%) for each of the genes was verified via standard dilution curves.

### Western blotting analysis

For western blotting, cell monolayers were lysed in lysis buffer, and then briefly sonicated, boiled, and centrifuged for 10 min at 4 °C. The concentration of protein was assessed with the Pierce BCA protein assay reagent (Thermo Fisher Scientific). The protein samples were subjected to sodium dodecyl sulfate-polyacrylamide gel electrophoresis and transferred onto Immobilon-P membranes (EMD Millipore, Billerica, MA, USA). A rabbit anti-mouse p53 polyclonal antibody (FL-393; Santa Cruz Biotechnology, Dallas, TX, US) or a mouse anti-human p53 monoclonal antibody (DO-1; Santa Cruz Biotechnology) were used as primary antibodies, followed by respective horseradish peroxidase-conjugated secondary antibodies. The antigen-antibody complex was visualized by an enhanced chemiluminescence substrate (GE Healthcare).

### Statistical analysis

All statistical analyses were performed using GraphPad Software package (GraphPad Software, La Jolla, CA, USA). Statistical analyses for *in vitro* experiments were conducted using one-way analysis of variance. The animal infection analysis was performed using the non-parametric Mann–Whitney U test. Mean values are shown in the figures. Statistical significance was established at *P* < 0.05.

## Additional Information

**How to cite this article**: Wang, S. *et al*. Tumor suppressor p53 protects mice against *Listeria monocytogenes* infection. *Sci. Rep*. **6**, 33815; doi: 10.1038/srep33815 (2016).

## Figures and Tables

**Table 1:** Primers used in this study.

Primer	Sequence (5′ to 3′)	Target gene
mp53 siRNA-F	GUACAUGUGUAAUAGCUCCTT	mouse p53
mp53 siRNA-R	GGAGCUAUUACACAUGUACTT	
hp53 siRNA-F	GACUCCAGUGGUAAUCUACTT	human p53
hp53 siRNA-R	GUAGAUUACCACUGGAGUCTT	
NC siRNA-F	UUCUUCGAACGUGUCACGUTT	
NC siRNA-R	ACGUGACACGUUCGGAGAATT	
mGAPDHRT-F	GCACAGTCAAGGCCGAGAAT	mouse GAPDH
mGAPDHRT-R	GCCTTCTCCATGGTGGTGAA	
mp53RT-F	GCATGAACCGCCGACCTATCC	mouse p53
mp53RT-R	CAGGGCAGGCACAAACACGAAC	
mTNFαRT-F	CATCTTCTCAAAATTCGAGTGACAA	mouse TNFα
mTNFαRT-R	TGGGAGTAGACAAGGTACAACCC	
mIL-6RT-F	GAGGATACCACTCCCAACAGACC	mouse IL-6
mIL-6RT-R	AAGTGCATCATCGTTGTTCATACA	
mIL-12RT-F	GGAAGCACGGCAGCAGAATA	mouse IL-12
mIL-12RT-R	AACTTGAGGGAGAAGTAGGAATGG	
mIL-18RT-F	CAGGCCTGACATCTTCTGCAA	mouse IL-18
mIL-18RT-R	TCTGACATGGCAGCCATTGT	
mIFNγRT-F	TCAAGTGGCATAGATGTGGAAGAA	mouse IFNγ
mIFNγRT-R	TGGCTCTGCAGGATTTTCATG	
mGBP1RT-F	GAGTACTCTCTGGAAATGGCCTCAGAAA	mouse GBP1
mGBP1RT-R	TAGATGAAGGTGCTGCTGAGGAGGACTG	
hGAPDH RT-F	CGGGAAGCTTGTGATCAATGG	human GAPDH
hGAPDH RT-R	GGCAGTGATGGCATGGACTG	
hp53 RT-F	AGGCCTTGGAACTCAAGGAT	human p53
hp53 RT-R	TGAGTCAGGCCCTTCTGTCT	
